# Lipid Metabolites Enhance Secretion Acting on SNARE Microdomains and Altering the Extent and Kinetics of Single Release Events in Bovine Adrenal Chromaffin Cells

**DOI:** 10.1371/journal.pone.0075845

**Published:** 2013-09-20

**Authors:** Virginia García-Martínez, José Villanueva, Cristina J. Torregrosa-Hetland, Robert Bittman, Ashlee Higdon, Victor M. Darley-Usmar, Bazbek Davletov, Luis M. Gutiérrez

**Affiliations:** 1 Instituto de Neurociencias de Alicante, Universidad Miguel Hernández, Consejo Superior de Investigaciones Científicas, Sant Joan d’Alacant, Alicante, Spain; 2 Department of Chemistry and Biochemistry, Queens College, CUNY, Flushing, New York, United States of America; 3 Department of Pathology, Center for Free Radical Biology, University of Alabama, Birmingham, Alabama, United States of America; 4 MRC Laboratory of Molecular Biology, Cambridge, United Kingdom; Vanderbilt University Medical Center, United States of America

## Abstract

Lipid molecules such as arachidonic acid (AA) and sphingolipid metabolites have been implicated in modulation of neuronal and endocrine secretion. Here we compare the effects of these lipids on secretion from cultured bovine chromaffin cells. First, we demonstrate that exogenous sphingosine and AA interact with the secretory apparatus as confirmed by FRET experiments. Examination of plasma membrane SNARE microdomains and chromaffin granule dynamics using total internal reflection fluorescent microscopy (TIRFM) suggests that sphingosine production promotes granule tethering while arachidonic acid promotes full docking. Our analysis of single granule release kinetics by amperometry demonstrated that both sphingomyelinase and AA treatments enhanced drastically the amount of catecholamines released per individual event by either altering the onset phase of or by prolonging the off phase of single granule catecholamine release kinetics. Together these results demonstrate that the kinetics and extent of the exocytotic fusion pore formation can be modulated by specific signalling lipids through related functional mechanisms.

## Introduction

The exocytic fusion of specialized vesicles releasing their content of neurotransmitters and hormones is the central event underlying the physiological function of neuronal and endocrine systems. Exocytosis is a multistep process mediated by a host of protein-protein and protein-lipid interactions which often include three SNARE (soluble N-ethylmaleimide sensitive factor attachment protein receptor) proteins: SNAP-25 and syntaxin-1 localized on the plasma membrane and synaptobrevin II on the vesicular membrane [1,2,3]. In essence, the dominant proteocentric concept suggests that fusion occurs between two passive membrane platforms that are disrupted and remodelled by catalytic proteins. Certainly, the SNARE proteins may provide the specificity required for vesicle docking and probably the basic machinery for membrane fusion [4], but it is also evident that lipids could be essential players or regulators of exocytosis [5,6,7]. In this respect, because membranes have to adopt different curvatures during fusion, it has been shown that cone-shaped lipids may favor the appropriate membrane geometry and thus can influence the membrane propensity to fuse [8].

In addition to this “structural role”, lipids may influence directly the fusion machinery by binding to individual or complexed SNAREs, and two important signalling lipids, AA and sphingosine, have become good examples for this type of regulation. For example, it has been suggested that AA, produced from phospholipid membranes by phospholipases, upregulates syntaxin-1, increasing the incorporation of this protein into fusogenic SNARE complexes [9,10,11]. On the other hand, sphingosine, the releasable backbone of sphingolipids, acts on vesicular synaptobrevin II, promoting the formation of the ternary complex and facilitating vesicle exocytosis in neuronal and endocrine systems [12]. Thus soluble lipids can affect different SNARE proteins to increase the number of ternary complexes and thereby enhance the secretory properties of neuroendocrine cells [11,12,13].

In the present work, using the high spatial and temporal resolution of total internal reflection fluorescence microscopy (TIRFM, [14]) and the possibility to analyse single granule fusion kinetics with amperometry [15], we report the effects of lipid metabolites on different exocytotic stages ranging from granule docking to the final fusion steps. Our results provide evidence that signalling lipids can affect docking and fusion steps in a different manner resulting in differences in the extent and kinetics of granule fusion events.

## Results

### FRET experiments suggest the “in vivo” molecular interaction between sphingosine and AA and SNARE microdomains

**Figure 1:**
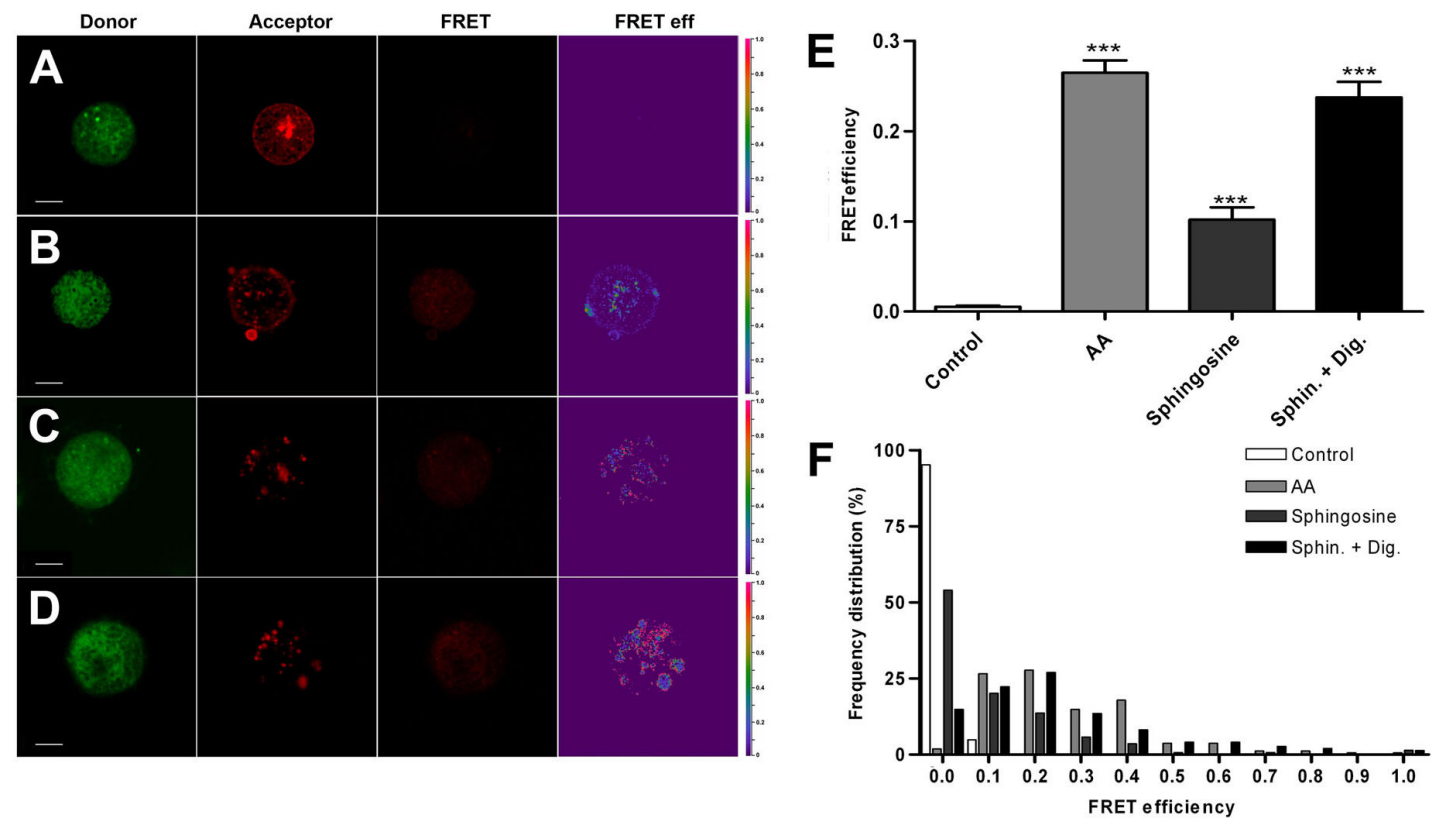
FRET signals suggest a direct molecular interaction between signalling lipids and SNARE microdomains. Chromaffin cells overexpressing DsRed-SNAP-25 were incubated in four different conditions: 1 µM BODIPY FL as the control condition (A), 1 µM AA-BODIPY (B), 1 µM sphingosine-BODIPY (C), and 1 µM sphingosine-BODIPY in the presence of 10 µM digitonin to permeabilize the cells (D). Representative images of four conditions are shown: (from left to right) the donor specimen, the acceptor specimen (each obtained with their optimal lasers), the FRET image (red channel illuminated with a 488 laser), and the image of apparent FRET efficiencies. To evaluate if the mean FRET signal was stronger in any of the four conditions, we measured the average intensity of several regions of interest (ROI), which is expressed as a percentage of the reference intensity under optimal conditions (E), subtracting cross-talk signals using factors obtained from donor only and acceptor only images according to Van Rheenen et al. [18]. The results indicate a significant apparent FRET efficiency when AA is used, which could be due to a direct interaction between AA and SNARE patches (p-value: <0.001 using ANOVA with Dunnett’s multiple comparison test). With respect to sphingosine, the results indicate a lower FRET efficiency (p-value: <0.001 compared to controls), when incubated with non-permeabilized cells, probably due to a poor diffusion into the cell. However, sphingosine treatment under digitonin permeabilization results in an increased apparent FRET signal (p-value: <0.001 compared to controls and to sphingosine in non permeabilized cells). Apparent FRET data distributions (F). N = 145 ROI from 11 BODIPY treated cells, N = 162 ROI from 24 AA-BODIPY treated cells, N = 139 ROI from 21 sphingosine-BODIPY treated cells and N = 148 ROI from 34 sphingosine-BODIPY FL treated and digitonin-permeabilized cells. Bin width 0.1 units. Bars: 3 μm.

In order to elucidate the mechanism utilized by signalling lipids to enhance the secretory response [11,12,13], we first tested the possible interaction of exogenous sphingosine and AA with the secretory machinery formed by SNAP-25-syntaxin microdomains in the plasma membrane of chromaffin [16] by using FRET sensitized emission experiments. These experiments were performed by incubation of cultured bovine chromaffin cells expressing SNAP-25-Ds-Red (FRET acceptor) with sphingosine or AA tagged with BODIPY (AA-BODIPY) as donor molecules. Two type of controls were used - soluble BODIPY by itself and sphingosine-BODIPY which cannot reach the interior of the cells [12]. FRET signals were measured as described before [17], following the method described by Van Rheenen et al. [18]. In these experiments the apparent FRET signals of individual SNAP-25-DsRed patches were expressed as the fluorescence at 488 nm referred to the obtained at the optimal excitation (543 nm), and channel crosstalk was taken in consideration by generation of calibration factors using acceptor and donor only references. Figure 1 shows fluorescence images from representative cells expressing SNAP-25-DsRed and incubated with 1 µM concentrations of soluble BODIPY (A), AA-BODIPY (B), sphingosine-BODIPY (C), or the same compound in the presence of 10 µM digitonin to permeabilize the cells (D). Figure 1 E shows that FRET signals (third row) were low in control conditions and resulted in low FRET efficiencies after crosstalk subtractions (fourth column), resulting in averaged values below 0.1 even in the case of sphingosine-BODIPY incubation in the absence of cell permeabilization. Incubation of the cells with AA or sphingosine with permealization resulted in stronger FRET efficiencies characterized by averaged values around 0.3, these values being statistically significant with respect to that obtained for soluble BODIPY (p<0.0001) or sphingosine-BODIPY in the absence of cell permeabilization (p<0.01). FRET efficiency value distributions revealed the displacement to 0.2-0.3 values indicating a clear FRET enhancement for SNAP-25-DsRed fluorescence when the cells are incubated either with AA-BODIPY or with sphingosine-BODIPY in permeabilized cells (Figure 1 F). These data support the hypothesis that signalling lipids can interact with SNARE microdomains in the plasmalemma of chromaffin cells.

### SNARE cluster motion is altered by signalling lipids

**Figure 2:**
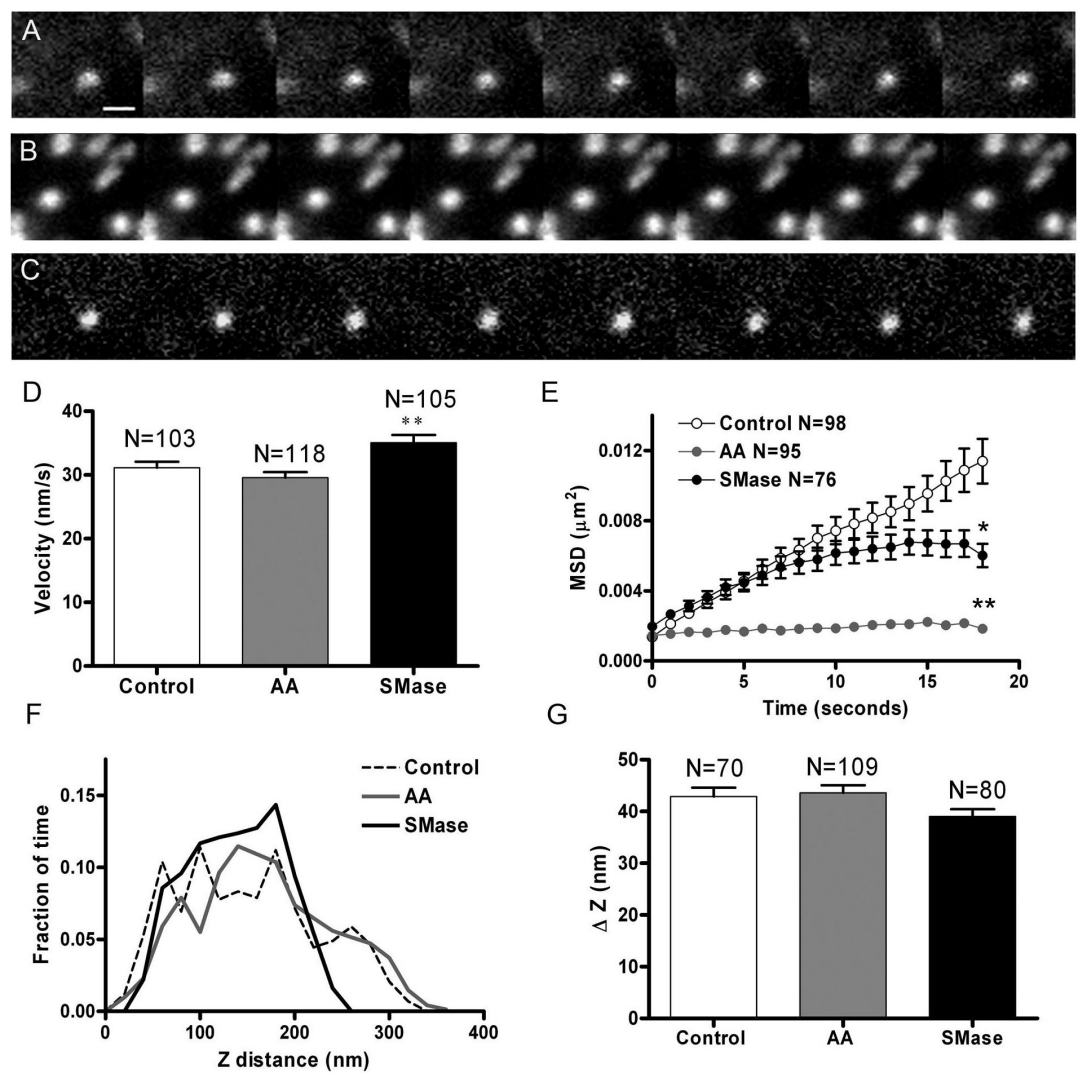
SNARE microdomain lateral motion is restricted in cells incubated with SMase and AA. The dynamics of the microdomains formed by expression of GFP-SNAP-25 were studied in time-lapse images taken at 1 s intervals in control chromaffin cells or cells treated with 100 μM AA or 1 U/ml SMase for 30 min. Shown are images separated by 5 s intervals of SNARE clusters in a control cell (A), a cell incubated with AA (B), and SMase (C). The microdomains display lateral mobility in the XY plane, which was studied by measuring the centroid coordinates, which were then used to calculate the average path speed (D) for the vesicles in control (N = 103), AA (N = 118), and SMase-treated cells (N = 105). E. Mean square displacement (MSD) vs time plot curves indicate a caged motion in the case of SMase-treated cells (downward curve) and almost immobile behaviour in AA-treated cells (flat curve, error bars smaller than symbols). The variations in the intensity of fluorescence indicate the different mobility in the Z plane for the same patches and were used to calculate Z distance distributions using 25 nm bin width (F), and increments (G). Bar represents 1 µm in A. *P<0.05 and **P<0.01 compared to controls using ANOVA with Dunnett’s multiple comparison test.

Next we studied how lipid metabolites could affect the dynamic behaviour of SNARE clusters on the plasma membrane. For this purpose, cultured chromaffin cells expressing GFP-SNAP-25 were observed under TIRFM and sequential images acquired at 1 s intervals, and then the images were subjected to particle tracking and the trajectories were analyzed to calculate cluster speed and diffusion parameters. Figure 2 depicts examples of time-lapse images of GFP-SNAP-25 clusters from control (Figure 2A), and cells treated with 100 µM AA (Figure 2B) or 1 U/ml of sphingomyelinase (SMase, Figure 2C) for 30 min [12,13]. SMase metabolizes sphingomyelin into ceramide which is then converted into sphingosine [12]. Under these conditions, SNAP-25 clusters show a motion characterized by short-range oscillations in the XY plane with average speeds around 30 nm/s. The average speed of the clusters did not change noticeably after cell treatment (Figure 2D) but the mean square displacements (MSD) vs time plot revealed that upon treatment with SMase the motion of the vesicles show a moderate restriction, displaying a downward saturating curve characteristic of caged behavior (Figure 2E, SMase curve). Interestingly, after treatment with AA, the SNAP-25 microdomain motion suffered major spatial restrictions as indicated by a flat MSD vs time curve, showing a great level of cluster immobilization (Figure 2E, AA curve). Assuming that SNAP-25 patch movement is governed by a single diffusion coefficient, its value can be obtained from the fitted slope (slope = 4x D) [19]. Thus the apparent coefficient of diffusion is 1.35 + 0.01 x 10^-4^ µm^2^/s for control SNAP-25 microdomains, whereas the diffusion coefficient was estimated to be 6.7 + 0.1 x 10^-5^ µm^2^/s for the clusters in SMase-treated cells, and reduced to 1.0 + 0.2 x 10^-5^ µm^2^/s in AA-treated cells, which is a ten-fold lower coefficient of diffusion compared to control cells. Further information comes from the analysis of SNAP-25 cluster motion in the Z plane. The maximum fluorescence intensity corresponding to the pixels defining SNAP-25 patches was studied (around 100 patches for every experimental condition), and then these fluorescence intensities were transformed into z distances using the equation defining the exponential evanescent field decay. GFP-SNAP-25 control patches moved distances ranging from 50 to 250 nm on the glass coverslip where the evanescent field was originated (Figure 2F). SNAP-25 patches in cells treated with SMAse displayed no significant change in the amplitude of the z motion (ΔZ, Figure 2G). It is important to note that AA treatment seems to slightly round the cells, and therefore the Z distance determinations could be affected by partial cell detachment from the glass surface.

### Signalling lipids affects chromaffin granule motion observed under TIRFM

**Figure 3:**
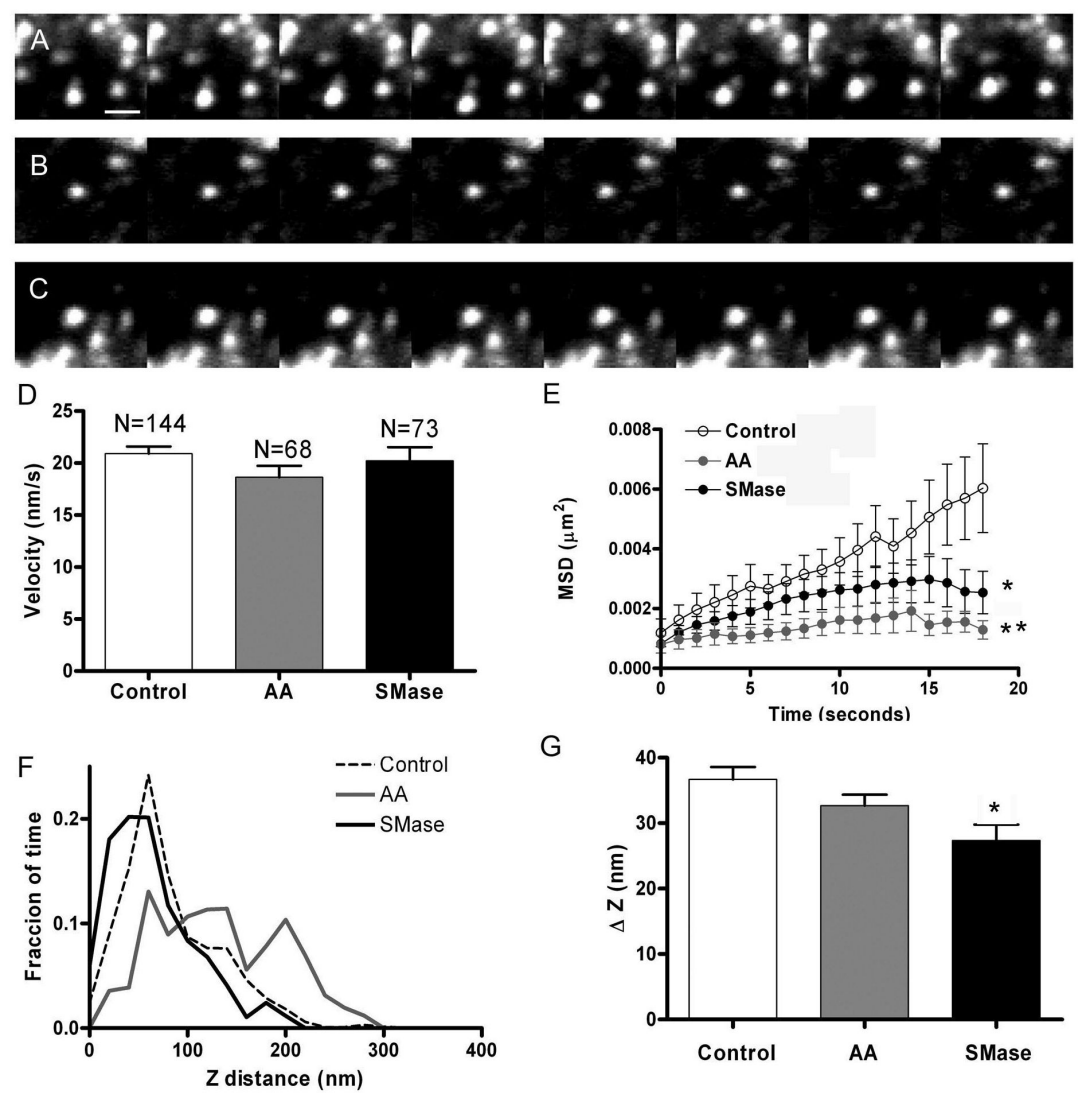
Granule motion is restricted after cell incubation with signalling lipids. Granule movement was studied in cells treated with AA and SMase as described in previous figures. Granules were labeled with acridine orange and motion was studied using TIRFM images acquired at 1 s intervals. Panels A, B, and C show images separated by 5 s intervals obtained in control (A), AA-treated cells (B), and SMase-treated cells (C). D. Averaged speed calculated for granules in control (n = 144), AA (n = 68), and SMase-treated cells (n = 73). **E**. Mean square displacement vs time for the granules indicate restriction in the mobility in SMase-treated cells and the high degree of immobilization found in the cells incubated with AA. **F**. Distribution of the Z distances corresponding to the granules in the indicated experimental conditions. 25 nm bin width. **G**. Calculation of the mean range of z displacement (ΔZ) obtained for granules in control, AA-treated cells, and SMase-treated cells. Bar represents 1 µm in A. * P<0.05 compared to control values using ANOVA with Dunnett’s post test.

Next we analyzed the behaviour of chromaffin granules in the immediate proximity of the plasma membrane where granule motion reflects the prefusion steps of the secretory cascade [16,20]. Again, chromaffin cells were treated with 1 U/ml SMase or 100 μM AA for 30 min and then control as well as treated cells were incubated with 2 μM acridine orange, an acidotropic dye allowing visualization of chromaffin granules and study of secretory kinetics under TIRFM [16]. Interestingly, addition of SMase and, even more so, AA resulted in a noticeable change in the motion of chromaffin granules. Example of the images acquired could be observed in Figure 3. Although the motion of the vesicles in these experimental conditions showed no changes in the average speed of these granules (Figure 3D), the mean square displacement vs time interval plots revealed a clear change in the restriction of vesicle movements after lipid treatment. Indeed, these plots exhibit lower asymptotic values than the curve characteristic of control non-treated vesicles (Figure 3E). Thus, the diffusion coefficient was calculated as 6.2 + 0.1 x 10^-5^ µm^2^/s for control vesicles (n = 145 vesicles from 29 cells), whereas the theoretical diffusion coefficient was estimated to be 4.0 + 0.1 x 10^-5^ µm^2^/s for the vesicles in SMase-treated cells (n = 73 vesicles from 22 cells), and 1.0 + 0.1 x 10^-5^ µm^2^/s for AA-treated cells (n = 52 vesicles from 18 cells). Moreover, from the asymptotic values we calculate that sphingosine treatment resulted in the restriction of the vesicles to a 210 nm radius. In the case of AA treatment, the vesicles were almost immobile and oscillated in a much narrower space. Again, an analysis of vesicle motion in the Z plane revealed a tendency to constrain the motion that was statistically significant in cells treated with SMase (Figure 3G). Taking into account the results obtained for the motion of SNAP-25 patches and the vesicles, it is clear that after cell treatment with AA, granules appear to be almost immobile, suggesting stronger docking [16,20]. On the other hand, SMase-treated cells present granules with a caged dynamics that could be assigned to tethered vesicles [20].

### Fusion of vesicles is accelerated in SMase treated cells observed by TIRFM

**Figure 4:**
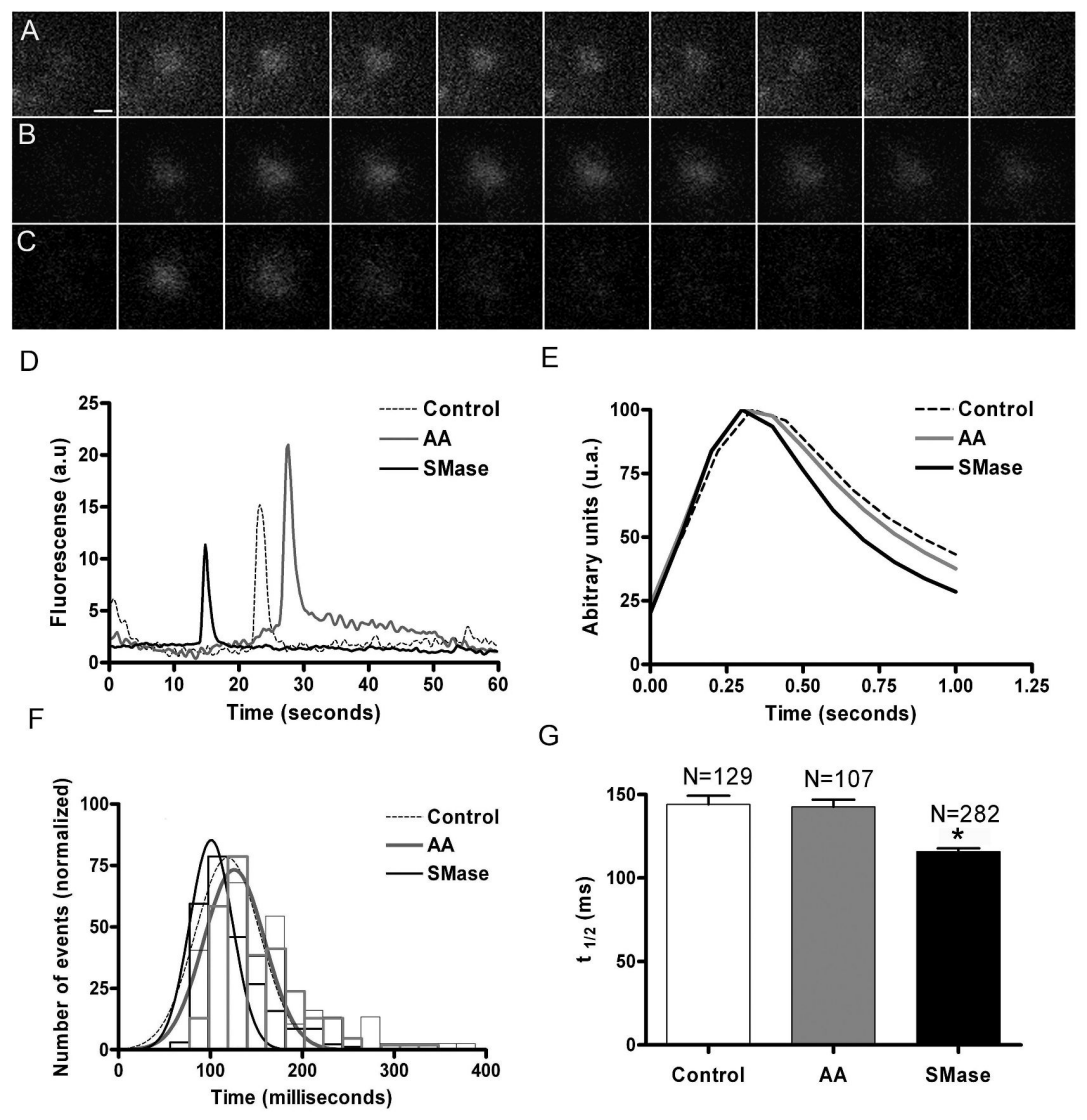
SMase treatment enhances the kinetics of chromaffin granule release visualized by TIRFM. Chromaffin cells were treated with AA and SMase and incubated with acridine orange as described in Methods. Cells were then subjected to 59 mM KCl depolarizations and vesicular fusions visualized in TIRFM images recorded at 20 ms intervals. A-C. Images of example fusions obtained in control (A), AA-treated cells (B), and SMase –treated cells (C). The mean intensity analysis of these fusion events resulted in secretory spikes as shown in panel D. Analysis of these optical events by the appropriate software resulted in averaged events in every experimental condition as shown in E.F. Distribution of the time at the half height (t_1/2_) corresponding to the peaks average, showing a displacement to lower values after the treatment with SMase. Bin with 20 ms. G. The average t_1/2_ parameter indicates narrower secretory spikes in SMase-treated cells. P<0.05 as compared to controls using the ANOVA with Dunnett’s post test.

Acridine orange-loaded vesicles can be used to study the kinetics of granule fusion in chromaffin cells [16]. In these experiments, cells were incubated with 2 µM acridine orange during 20 min and then were visualized using TIRFM. The laser intensity was low enough to prevent light-induced exocytosis [21], and granule fusion elicited by 59 mM KCl solution was observed as green flashes due to acridine orange neutralization upon exocytosis, recorded at 20 ms intervals (Figure 4A-C). These time-lapse images could be used to integrate the image intensity corresponding to the fusion of individual vesicles resulting in spikes resembling those characteristic of amperometric events (Figure 4D). These spikes were analyzed with programs developed for amperometry to obtain an averaged spike for secretory events (Figure 4E). This analysis revealed that, on average, the “optical” secretory spikes from cells treated with SMase are narrower that those observed in control as well as in cells treated with AA. This is further supported by measurement of kinetic parameters such as the time at the half height (t_1/2_) for more than 100 events in every experimental condition, showing distributions (Figure 4F) shifted to lower t_1/2_ values in the case of SMase treatment. In fact, the averaged t_1/2_ was decreased by 20% when compared to that obtained in control and AA-treated cells (Figure 4G, p <0.01). These results indicate that upon treatment with SMase, release of granule content tends to occur at a faster rate when compared with that obtained in control or AA-treated cells, suggesting again that these signalling lipids affect single vesicle exocytosis in a different manner.

### Amperometry evidence for altered properties of single granule release

**Figure 5:**
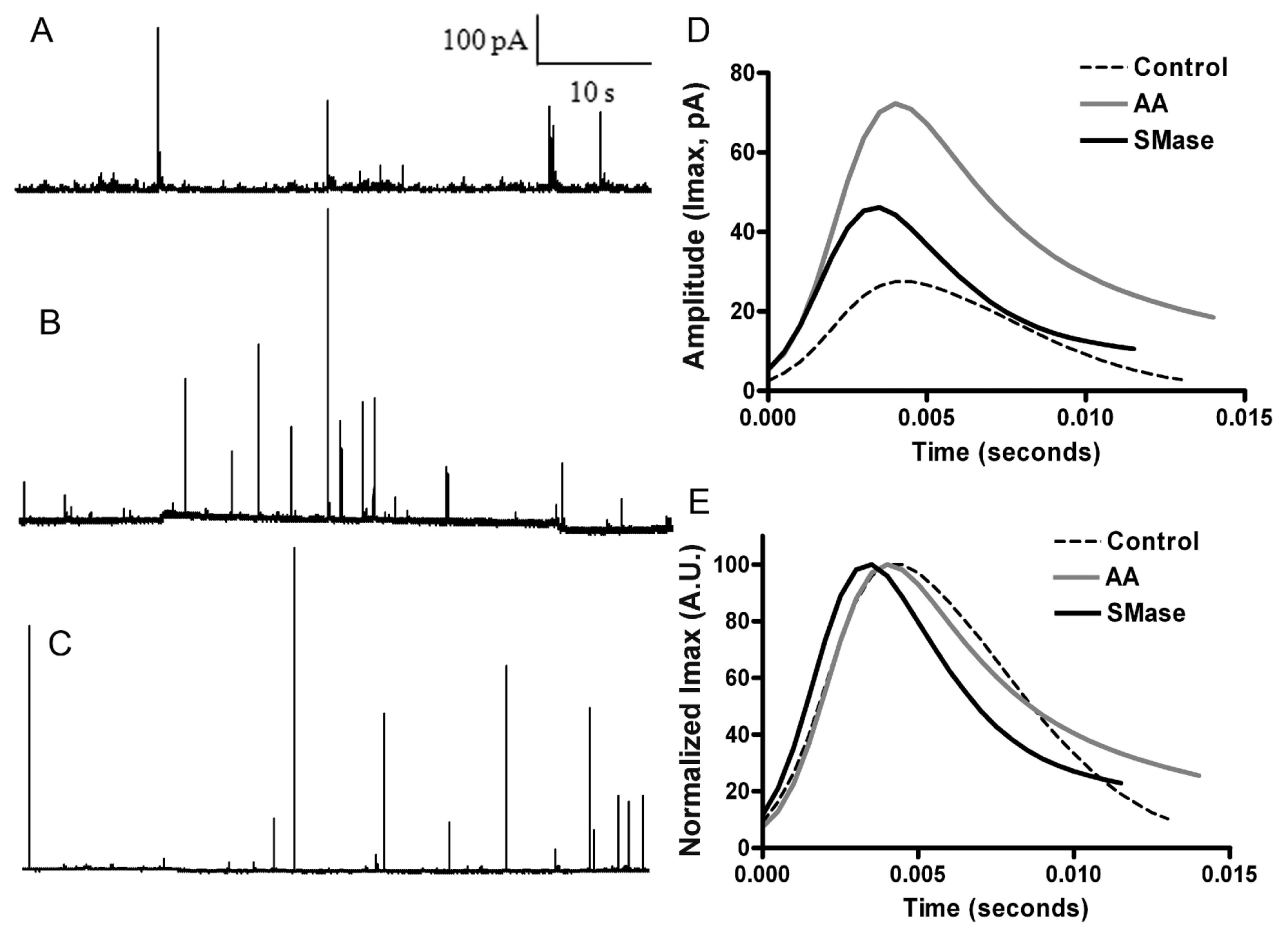
Effect of lipid metabolites on the amperometric granule release events. Depicted are amperometric recordings obtained from 59 mM KCl depolarizations in control (A), AA-treated cells (B), and SMase-treated cells. D. Amperometric fusion events were analyzed and pooled to generate the averaged amperometric spikes from control (N = 1262 events), AA (N = 828), and SMase-treated cells (N = 854). For kinetic studies the normalized spikes are compared to show an evident reduction in the t_1/2_ parameter in SMase-treated cells (E). Evidently, secretory events exhibit higher amplitude in the cells treated with signalling lipids.

The optically-based studies described above strongly suggest that signalling lipids affect the final events of vesicle exocytosis but a detailed description of such alterations is best achieved using amperometry. Experiments were performed using AA and SMase in the conditions described above, and amperometric traces were obtained in response to 59 mM KCl depolarizations. Representative examples are depicted in Figure 5, which shows that signalling lipids increase the amplitude of the individual spikes. An exhaustive analysis of amperometric spike characteristics using the Quanta Analysis program [22] gave average spikes from more than 800 amperometric events recorded from cells obtained in at least 3 separate culture preparations. As observed in Figure 5D, incubation with both SMase and more evidently with AA resulted in a higher amplitude (Imax) averaged spike as compared with the control average event. In addition, when these averaged spikes are normalized for intensity it becomes clear that SMase treatment results in narrower spikes compared to AA and control spikes (Figure 5E), suggesting accelerated fusion kinetics.

**Figure 6:**
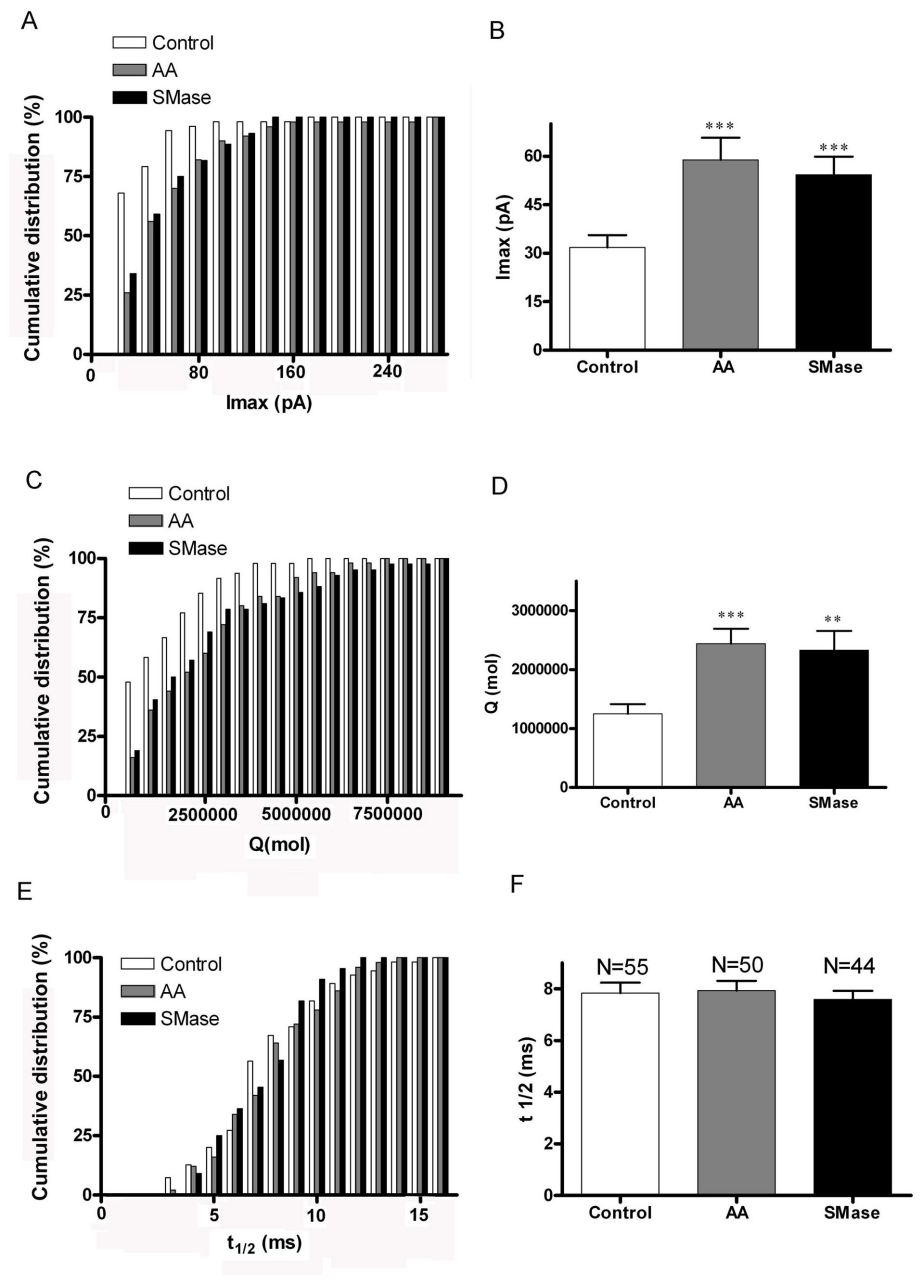
Signalling lipids dramatically enhance the amount of catecholamines released by event. Main amperometric parameters of the secretory spikes obtained from control (N=55 cells), as well as AA-treated cells (N=50) and SMase-treated cells (N=44). This analysis was performed by averaging the spikes obtained in every individual cell. A. Cumulative distribution of the Imax values averaged for individual cells. 20 pA bin width. B. The mean amplitude is enhanced by 2-fold in AA-treated cells and by 1.7-fold in SMase treated cells. C. Cumulative distribution of the averaged amount of catecholamine molecules released per event obtained for individual cells. 500.000 molecules/event bin width. D. Consequently, the mean value of released molecules per event increased by 2.1-fold in AA-treated cells and by 2-fold in cells incubated with SMase. E. Cumulative distribution of the t_1/2_ parameter averaged for individual cells. 1 ms bin width. F. Mean t_1/2_ parameter was slightly reduced with SMase-treatment (not significant at the statistical level. **P<0.01 and ***P<0.001comparing to control according to ANOVA with Dunnett’s test for multiple comparisons.

**Figure 7:**
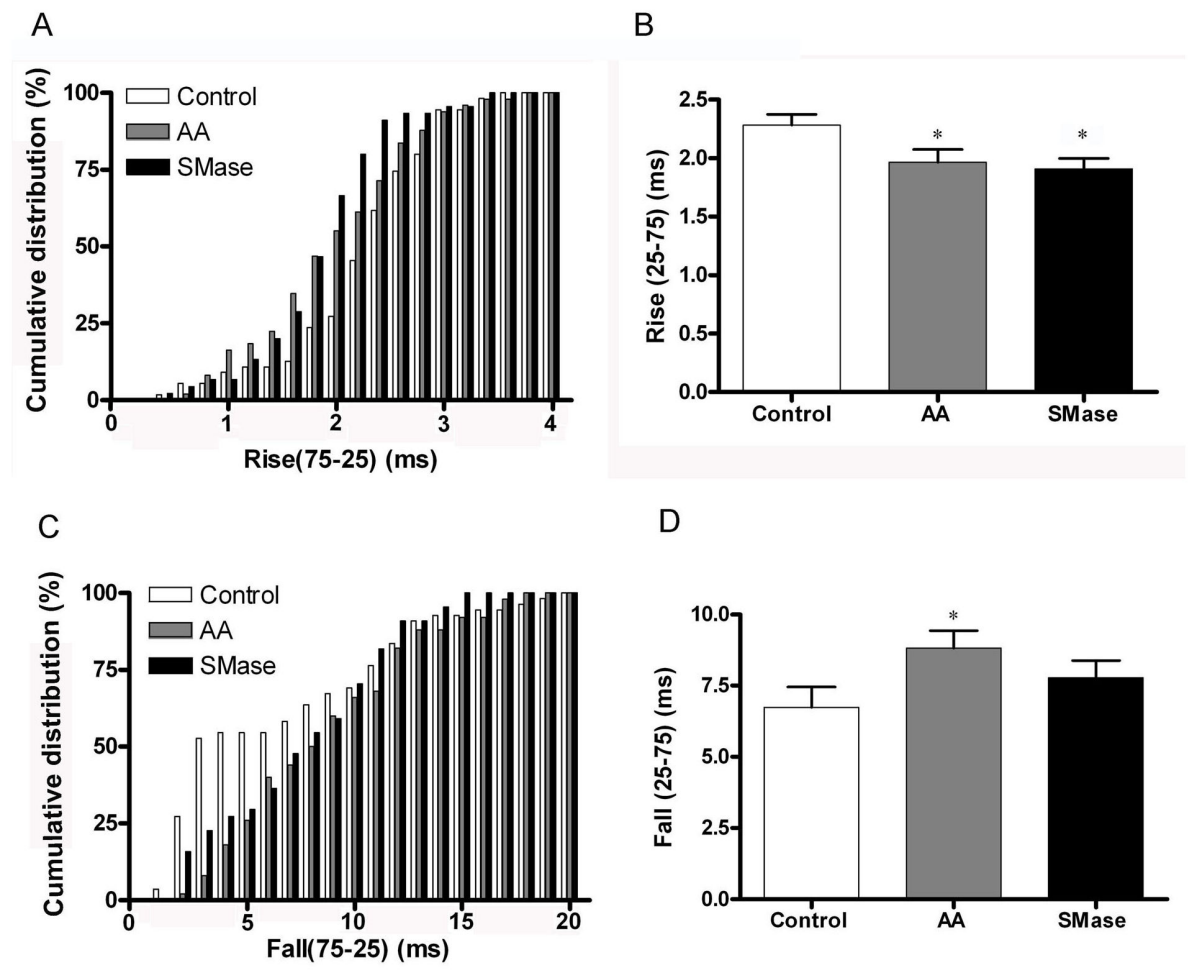
Signalling lipids affect the kinetics of catecholamine release from individual granules. Analysis of the kinetic parameters of amperometric spikes for the same number of cells indicated in the previous figure. A. Cumulative distribution of the onset rise parameter (time corresponding to the amplitude rise between the 25 and 75% of the maximal amplitude) for the different experimental conditions showing a displacement to lower values in response to lipid metabolites. 0.2 ms bin width. B. The average values are reduced after both treatments in a similar proportion (20%). C and D. Analysis for the offset parameter corresponding to the decaying phase of amperometric events. AA treatment affects this decaying phase, prolonging catecholamine release. Bin width 1 ms. *P<0.05 comparing to control according to ANOVA with Dunnett’s test for multiple comparisons.

A further quantitation of these differences based on pooling the spikes for individual cells and the study of the cumulative distributions corresponding to the different amperometric parameters (Figure 6) shows that AA-treated cells are characterized by a 2-fold higher mean amplitude and a wider distribution compared to control (Figure 7) whereas SMase treatment resulted in a 1.7-fold increase (Figure 6A). Interestingly, the amount of catecholamines released per individual event was increased in similar proportions, reaching a 2.2-fold increase with AA treatment and a 1.8-fold rise after the addition of SMase. However, overall kinetics measured up to the point of the half-height amplitude seems to be relatively unaffected by sphingolipid metabolites or AA treatment and resulted in no significant changes (Figure 6F). A detailed study of the spike onset characteristics measured by calculating the time comprised between the 25 and 75% of the rise phase of spikes shows that both lipids are able to statistically increase the speed of fusion pore opening (Figure 7A and B). An exhaustive analysis of the falling phase of the spikes revealed that AA modifies the decaying phase, prolonging the release of catecholamines, whereas SMase induced only a minor modification of this kinetic parameter (Figure 7C and D). These results imply that the main effect caused by sphingosine production is to accelerate the rate of fusion pore opening whereas AA treatment also prolongs the time of full aperture, increasing drastically the amount of catecholamines released per event.

## Discussion

### Signalling lipids modulate exocytotic stages and the properties of single release events during exocytosis

The present work reports several findings important for modulation of exocytosis: first, signalling lipids can interact at the molecular level with SNARE microdomains and change their dynamic properties; second, these interactions differentially affect the secretory pathway at the level of granular tethering and docking; and finally, lipids can cause alterations of the release of catecholamines from individual granules, resulting either in acceleration of the rate of fusion pore dilation or prolongation of the time of aperture of the fusion pore. Previously, a positive role for sphingosine and AA in exocytosis was predicted based on their ability to promote formation of SNARE complexes in neurons [9,11,23], and the functional logical consequence of SNARE’s increased ability to form complexes is an increase in the secretory properties of neurons and neuroendocrine cell models [11,12,13]. Our data indicate, that lipid metabolites can interact with the microdomains of SNARE proteins and, more importantly, that this has consequences on the dynamic behaviour of these microdomains. The lateral mobility characteristics of chromaffin granules studied under TIRFM have been useful to distinguish between docking and priming steps [20], Furthermore, the motion of these docked granules is naturally linked to the dynamics of SNARE microdomains due to both tethering and docking [16,24]. Our study now demonstrates that lipid metabolites reduce the mobility of both plasma membrane SNARE clusters and vesicles to a different extent. SMase treatment results in favoring the caged motion of the vesicles, a behaviour that has been associated with vesicle tethering [20]. This could be explained by the increased ability of synaptobrevin to engage syntaxin-SNAP-25 heterodimers, a molecular event associated with vesicular tethering. Instead AA, by increasing the number of syntaxin/SNAP-25, could have a more pronounced effect over SNARE cluster motion, causing stronger immobilization [20]. Obviously, other molecular events involving both proteins and lipid bilayer properties cannot be excluded at this moment. Regardless of the detailed mechanisms, our data show that signalling lipids affect different steps of the exocytotic mechanism.

The profile of the amperometric spikes can reveal the kinetic characteristics of the fusion pore opening, aperture time and closing [25,26,27]. Our results indicate that both SMase and AA affect the initial rate of expansion of the fusion pore as indicated by lower values in the t_25-75_ rise parameter. Most importantly, AA prolongs the time of fusion pore opening, resulting in drastic enhancement of neurotransmitter release, up to 2-times the values compared to control conditions. These results imply that neuroendocrine single vesicle release even with high potassium-driven depolarizations is actually suboptimal (e.g., ‘kiss and run’ mechanism) and signalling lipids appear to facilitate the transition to full fusion mechanism. The magnitude of this modulation is impressive and much more evident than the modification of single fusion kinetics found when mutating SNARE proteins such as SNAP-25 and synaptobrevin [25,28,29]. Taking together, our results reveal that signalling lipids such AA and sphingosine can modulate the extent and kinetics of single vesicle release in a specific manner. These changes are more dramatic compared to those induced by structural plasma membrane phospholipids such phosphatidylcholine, phosphatidylethanolamine, phosphatidylserine, or sphingomyelin [30], which cause only moderate alterations in the rate of neurotransmitter release but not in the quantal size.

The main objective of the present work was to use chromaffin cells as a test system to study the mechanisms utilized by signalling lipids to enhance secretion. We demonstrated that lipid metabolites through possible interactions with SNARE microdomains could regulate neurotransmitter release far more directly than traditionally thought, by acting as dynamic rather than passive components of the vesicle fusion machinery. It has been demonstrated that AA is released from chromaffin granule membranes during secretion [31] and production of sphingolipid derivatives regulates a vast variety of physiological processes [32]. Further studies should look into the transient generation of signaling lipids during chromaffin cell stimulation as well as a more detailed characterization of the molecular mechanisms involved.

## Materials and Methods

### Fluorescent lipids

Sphingosine-BODIPY was prepared according to [33,34]. AA-BODIPY was prepared as in [35]. BODIPY-Texas-Red ceramide was purchased from Life Technologies (Carlsbad, CA, USA).

### Chromaffin cell preparation and culture

Bovine Adrenal glands were obtained from the local slaughterhouse (Matadero Orihuela SA, Orihuela, Alicante, Spain). We obtained permission from this company for the use of adrenal glands in research) and chromaffin cells were isolated from bovine adrenal glands by collagenase digestion as described before [36]. Briefly, cells were further purified from the debris and erythrocytes by centrifugation on Percoll gradients and maintained as monolayer cultures in Dulbecco’s modified Eagle’s medium (DMEM) supplemented with 10% fetal calf serum, 10 μM cytosine arabinoside, 10 μM 5-fluoro-2`-deoxyuridine, 50 IU/ml penicillin, and 50 μg/ml streptomycin. Finally, cells were harvested at a density of 150,000 cells/cm^2^ in 35-mmPetri dishes (Costar), and were used between the third and sixth day after plating.

### Generation of GFP construct with SNAP-25 and cell transfection

The pEGFP-C3 expression vector (Clontech, Palo Alto, CA) encodes a red-shifted variant of wild-type GFP [37]. The cDNA corresponding to the SNAP-25a isoform [38] was cloned into the XhoI and BamHI sites of pEGFP-C3 to express this protein fused in-frame at the C-terminus to EGFP (construct GFP-SNAP-25). The amplified DNA carried an internal HindIII site, close to the 5’-end, and the previously mentioned BamHI site at the 3’-end. These enzymes were used to substitute the original SNAP-25 sequence by the modified one in the GFP-SNAP-25 vector. Chromaffin cells were transfected using the Amaxa basic nucleofector kit for primary mammalian neuronal cells according to the manufacturer’s instructions (Program O-005, Amaxa GmbH, Koehl, Germany).

### Total internal reflection fluorescence microscopy (TIRFM) studies of GFP-SNAP-25 dynamics, vesicular motion, and fusion

A through-the-lens TIRFM system was configured using the Olympus IX-71 inverted microscope indicated above with a 100x PlanApo 1.45 N.A. Olympus TIRFM objective. Epifluorescence and laser illumination (488 nm argon ion 40 mW or 543 nm He/Ne 10 mW: Melles Griot, Carlsbad, CA, USA) was selected using an Olympus TIRFM IX2-RFAEVA combiner system, modifying the angle of laser incidence. Fluorescence emission was split using an Optosplit II system (Cairn Research Ltd, Favershaw, UK) equipped with GFP and rhodamine filter sets. The separated images were simultaneously acquired at 20 ms per frame using an Electron Multiplier CCD cooled camera (C9100-02 model, Hamamatsu photonics, Shizuoka Pref, Japan) and stored in an IBM-compatible PC. TIRFM calibration was performed using 100 nm fluorescent beads (Life Technologies). The fluorescence intensities were determined at different vertical planes with step lengths of 100 nm using the motorized system mounted on the microscope, and the image was obtained for both epifluorescence and TIRFM. The depth of penetration for the evanescent field was estimated as ~200 nm (1/e depth of 180 ± 16 nm), permitting visualization of the static beads adhered to the coverslip. In contrast, beads in suspension undergoing random movement were infrequently seen in TIRFM, and the vast majority was visualized by epifluorescence. In vesicle fusion experiments, granule labelling was performed with 2 μM acridine orange. In the latter, the granules were assessed by the red acridine orange fluorescence in mature acidic vesicles whereas their fusion was followed by the green flashes produced after matrix neutralization during exocytosis according to procedures described previously [16] Images were processed using the ImageJ program with Plugins for particle centroid tracking, ROI measurements, image average, multiple channel image comparison, and co-localization analysis [39]. Further analysis such as the MSD determinations according to Qian et al. [19] and z distances according to Johns et al. [40] were performed using home-made macros for Igor Pro (WaveMetrics Inc, Lake Oswego, OR, USA). For the analysis of fusion events in acridine orange labelled vesicles, the green channel images taken at 20 ms intervals that showed fusion flashes were subjected to maximal intensity determination (see Figure 3) and transferred to Igor Pro. Fusion events were analyzed using software developed for amperometric detection of exocytotic events (Quanta analysis [22]). Kinetic parameters such as the time at the half-height amplitude (t_1/2_) were obtained for hundreds of fusion events and are represented as distributions. The fusion peak shape was averaged for individual cells and studied for statistical variations. Controls were taken in the presence of 0.1% DMSO, final concentration of the solvent used to prepare AA. SMase was prepared in water and the cells treated with this enzyme were also treated with 0.1% DMSO. All the functional experiments were performed at 21-22 °C.

### FRET study of sphingosine and AA interaction with SNARE microdomains

FRET sensitized emission experiments were performed in cells expressing SNAP-25-Ds-Red (FRET acceptor) with sphingosine or AA labelled with BODIPY as a donor molecule (1 µM concentration). FRET signals were measured in a Leica TCS2 confocal microscope using software designed to take into account calibrations with donor only and acceptor only samples as described before [17], and according to the method described by Van Rheenen et al. [18]. In these experiments the apparent FRET signals of individual SNAP-25-DsRed patches were expressed by the equation:

E_A_(i)= (B-Axb-Cx(c-axb))/C

Where the apparent FRET efficiency (E_A_(i)) is a function depending on the intensities of the donor, FRET and acceptor channels (A,B,C), as well as the calibration factors obtained from donor only and acceptor only samples (a,b, and c). These calibration samples were measured every experimental day and the parameters averaged from at least 10 different cells. The FRET program keeps constant the parameters for laser intensity and channel acquisition and we choose these parameters avoiding intensity saturation.

In these experiments we use two type of controls; incubating the cells with free BODIPY or sphingosine-BODIPY that barely permeates the plasma membrane [12].

### Amperometric determination of exocytosis

To study secretory activity from control non-transfected and fluorescence-emitting cells expressing the different constructs, we replaced the culture media with Krebs/HEPES, (K/H) basal solution with the following composition (in mM): NaCl (134), KCl (4.7), KH_2_PO_4_ (1.2), MgCl_2_ (1.2), CaCl_2_ (2.5), glucose (11), and Hepes (15), and the pH was adjusted to 7.4 with a NaOH solution. Carbon-fiber electrodes insulated with polypropylene and with 14 µm diameter tips were employed to monitor catecholamine release from individual chromaffin granules in cells under superfusion [41]. Electrodes were positioned in close apposition to the cell surface using high precision hydraulic micromanipulation and assessing cell membrane deformation with an Axiovert 135 inverted-stage microscope (Zeiss, Oberkochen, Germany) mounting Hoffman optics (Modulation Optics, Greenvale, NY). Electrical connection was accomplished with mercury. An amperometric potential of +650 mV versus an Ag/AgCl bath reference electrode was applied using an Axopatch 200A amplifier (Axon Instruments, Foster City, CA, USA). Current product of the catecholamine oxidation was digitized with an A/D converter and recorded at 400 μs/point using the program Clampex program (Axon) running on a PC computer. Experiments were performed in cells stimulated by superfusion with depolarizing 59 mM high potassium during 40 s (obtained by replacing isosmotically NaCl by KCl) and applied through a valve-controlled puffer tip commanded by the acquisition software and located near the studied cells. Curve fitting to non-linear models provided or implemented in software (Igor Pro and Graphpad Prism) was used to analyze data. Individual spike characteristics were studied using the Quanta program mentioned above, allowing for peak detection, integration, and kinetic parameter calculations. After acquisition at 2 KHz, only well- defined narrow peaks with amplitudes higher than 5 pA were taken to build event histograms, ensuring that the vesicle fusions analyzed were produced in the electrode proximity and, therefore, fully oxidation of released catecholamines was accomplished. Cell to cell variations were alleviated by using the same electrodes for measurements in control and lipid treated cells. During analysis recordings were filtered using binomial filters provided by the program and ensuring that the peak amplitude or kinetics was not affected. The rate of filtering estimated by the program is equivalent to 400 Hz low frequency filter. Data was analyzed using distributions for the different parameter obtained from individual cells.

Statistical significance was studied using ANOVA with Dunnett’s test for multiple comparisons implemented in GraphPad Prism 4.0.
